# Metagenomic next-generation sequencing targeted and metagenomic next-generation sequencing for pulmonary infection in HIV-infected and non-HIV-infected individuals

**DOI:** 10.3389/fcimb.2024.1438982

**Published:** 2024-08-19

**Authors:** Luyao Sun, Kaiyu Zhang, Yong Liu, Lihe Che, Peng Zhang, Bin Wang, Na Du

**Affiliations:** ^1^ Department of Infectious Diseases, The First Hospital of Jilin University, Changchun, China; ^2^ Genetic Diagnosis Center, The First Hospital of Jilin University, Changchun, China

**Keywords:** bronchoalveolar lavage fluid, metagenomic next-generation sequencing (mNGS), targeted metagenomic next-generation sequencing (tNGS), HIV infection, pathogens

## Abstract

**Background:**

When individuals infected with human immunodeficiency virus (HIV) experience pulmonary infections, they often exhibit severe symptoms and face a grim prognosis. Consequently, early, rapid, and accurate pathogen diagnosis is vital for informing effective treatment strategies. This study aimed to use metagenomic next-generation sequencing (mNGS) and targeted mNGS (tNGS) to elucidate the characteristics of pulmonary infections in HIV and non-HIV individuals.

**Methods:**

This study enrolled 90 patients with pulmonary infection at the Department of Infectious Diseases of The First Hospital of Jilin University from June 2022 to May 2023, and they were divided into HIV (n=46) and non-HIV (n=44) infection groups. Their bronchoalveolar lavage fluid (BALF) was collected for mNGS analysis to evaluate the differences in pulmonary infection pathogens, and tNGS detection was performed on BALF samples from 15 HIV-infected patients.

**Results:**

A total of 37 pathogens were identified in this study, including 21 bacteria, 5 fungi, 5 viruses, 5 mycobacteria, and 1 mycoplasma. The sensitivity of mNGS was 78.9% (71/90), which is significantly higher than that of conventional methods (CTM) (39/90, P=1.5E-8). The combination of mNGS with CTM can greatly enhance the sensitivity of pathogen detection. The prevalence of *Pneumocystis jirovecii* (82.6% *vs.* 9.1%), cytomegalovirus (CMV) (58.7% *vs.* 0%), and Epstein-Barr virus (EBV) (17.4% *vs.* 2.3%) was significantly higher in the HIV infection group than in the non-HIV infection group (*P*<0.05). Although no statistically significant difference was observed, the detection rate of *Mycobacteria* was higher in HIV-infected patients (17.4%) than in the non-HIV group (6.8%). Furthermore, the tNGS results of BALF from 15 HIV-infected patients were not entirely consistent with the mNGS results., and the concordance rate of tNGS for the detection of main pathogens reached 86.7% (13/15).

**Conclusion:**

Next-generation sequencing (NGS) can accurately detect pathogens in the BALF of patients with pulmonary infection. The sensitivity of tNGS is comparable to that of mNGS. Therefore, this technique should be promoted in the clinic for better patient outcomes.

## Introduction

Pulmonary infection is one of the most common causes of fatality among humans due to its rapid onset, quick progression, and multiple comorbidity (Kalil et al., 2016). The World Health Organization estimates that more than 20 million people die of pneumonia annually (McAllister et al., 2019). It is caused by many pathogens, including bacteria, viruses, and fungi, and is more likely to occur in immunocompromised patients, especially human immunodeficiency virus (HIV)-infected individuals (Cribbs et al., 2020). Such cases may present severe manifestation and worse outcomes, thus requiring early, rapid, and accurate detection of pathogens to guide an effective treatment strategy and improve the prognosis. However, conventional methods (CTM), such as culture, sputum smear, and molecular assays, have low diagnostic efficiency of only 30-40% (Afsar et al., 2018; Phetsuksiri et al., 2020). So, clinicians frequently prescribe empirical anti-infective treatment based on clinical manifestations and imaging data. Nevertheless, there are significant differences in the types and numbers of pathogens between HIV-infected and non-HIV-infected people (Tan et al., 2023).

Metagenomic next-generation sequencing (mNGS) has recently been applied for diagnosing gene mutations as well as many infectious diseases like pulmonary infection (Pourahmadiyan et al., 2022; Kullar et al., 2023). This technique has a significantly higher detection speed over CTM and can perform high-throughput sequencing analysis of DNA or RNA using specimens (Schlaberg et al., 2017). By comparing the sequence data with the reference genome or a particular database, clinicians can identify the variation and quantity of various microorganisms as well as drug-resistance genes (Ávila-Ríos et al., 2020). Many studies have reported the high sensitivity of mNGS for detecting pathogens (Ávila-Ríos et al., 2020; Zhang et al., 2022; Kullar et al., 2023; Wu et al., 2023). Nevertheless, the obtained specimens can affect the accuracy of mNGS. Sputum and throat swabs are not ideal specimens for detection tests because they are more likely to be contaminated, and they carry fewer germs than bronchoalveolar lavage fluid (BALF) (Wu et al., 2023). Despite BALF being more difficult to obtain than sputum and throat swabs from patients with pulmonary infection, it is a more reliable and acceptable sample for mNGS analysis (Zhang et al., 2022). In clinical applications of mNGS, following the initial mNGS detection, the targeted metagenomic next-generation sequencing (tNGS) method has gradually emerged. mNGS is relatively expensive and may be affected by human genomic interference. tNGS, on the other hand, combines multiplex PCR amplification with high-throughput sequencing, focusing on detecting known pathogenic microorganisms and drug resistance genes in the samples. tNGS reduces detection costs and increases the sensitivity of pathogen detection, which can simultaneously detect DNA and RNA, shorten detection time, and is characterized by high cost-effectiveness and customizability.

Currently, there are few researches on the characteristics of pathogens among HIV-infected patients complicated with pneumonia. This study retrospectively enrolled HIV-infected and non-HIV-infected patients, evaluated the diagnostic value of mNGS and tNGS in the diagnosis of pulmonary infections, and summarized the pathogen profiles in such cases. Additionally, the performance of CTM and mNGS in diagnosing pulmonary infections was also summarized.

## Methods

### Study subjects

This study recruited 90 patients diagnosed with lower respiratory tract infection at the Department of Infectious Diseases of The First Hospital of Jilin University from June 2022 to May 2023. There were 46 patients who tested positive for HIV infection and 44 patients who tested negative for HIV infection. All patients underwent HIV antigen-antibody testing. For patients with positive HIV screening antibodies, a confirmatory Western blot (WB) assay was performed for confirmation. The detailed demographic information, clinical data, and sequencing results of the patients were obtained from the electronic medical records. The research protocol was approved by the ethics committee of our hospital (No. 2021-022-01). Each individual provided written informed consent.

### BALF extraction and mNGS analysis

Before extracting the BALF, the routine preoperative preparation was performed, with 2% lidocaine (15-20 mL) being used for nebulization anesthesia. The BF-260 electronic bronchoscope (Olympus, Japan) was applied to extract the BALF with 100-150 mL of sterile physiological saline at 37°C. Then the specimen was placed in a bottle coated with silicone.

And then, 1 mL of BALF sample was centrifuged at 12,000 rpm for 5 min to collect the pathogens and human cells. Next, 50 μL of precipitate underwent depletion of host nucleic acids using 1 U of benzonase and 0.5% Tween 20 at 37°C for 5 min. Subsequently, total nucleic acids were extracted using a QIAamp UCP pathogen minikit (Qiagen, Germany). To generate the library, 30 μL of the nucleic acid eluate was used for DNA fragmentation, end repair, adapter ligation, and polymerase chain reaction (PCR) amplification using a Nextera DNA Flex kit (Illumina, San Diego, CA, USA). A Qubit dsDNA HS assay kit was used to measure the library concentration. An Agilent 2100 Bioanalyzer (Agilent Technologies, Santa Clara, CA, USA) was applied to assess the library quality with a high-sensitivity DNA kit. Besides, peripheral blood mononuclear cell samples from healthy donors were prepared using the same protocol, and sterile deionized water was extracted alongside the specimens to serve as a non-template control (NTC) (Deng et al., 2022; Tao et al., 2022). The pooled libraries were sequenced on a Nextseq 550 sequencing system (Illumina, San Diego, CA, USA) with a single-end sequencing kit.

### tNGS pipeline

Based on multiplex PCR and mNGS, in-house tNGS panel was designed to detect 273 pathogens causing infections in different systems according to the public pathogen databases and the published studies. The 273 pathogens included 113 bacteria, 47 fungi, 101 viruses, and 12 parasites. After DNA extraction, multiplex PCR with the designed primers was used to construct libraries. Library concentrations were quantified using Qubit 4.0. Illumina NextSeq platform was used for high-throughput sequencing.

### Bioinformatics analysis

Trimmomatic software was used to remove the low-quality reads, adapter contamination, duplicate reads, as well as reads shorter than 35 bp. Low-complexity reads were also removed. Human sequence data were identified and excluded by mapping to a human reference genome (hg38) with the Burrows–Wheeler Aligner (BWA) software. The remaining sequencing information was aligned with the most recent databases for bacteria, viruses, fungi, and protozoa provided by the National Center for Biotechnology Information. Reads that met the criteria for being considered unique were those with alignment lengths greater than 80%, sequence identities over 90%, and suboptimal to optimal alignment score ratios less than 0.8.

### Criteria for a positive mNGS/tNGS result

The specifically mapped read number (SMRN) of each microbial taxonomy was normalized to the SMRN per 20 million total sequencing reads to give the standardized SMRN (SDSMRN). The criteria for reporting the positive mNGS/tNGS results were as follows (Qin et al., 2021): (1) SDSMRN≥3 for bacteria (mycobacteria excluded); (2) SDSMRN≥3 for fungi/DNA viruses; (3) SDSMRN≥1 for RNA viruses; (4) SDSMRN≥100 for parasites; (5) SDSMRN≥3 for *Mycoplasma/Chlamydia* spp.; (6) SDSMRN≥1 (or SDSMRNG≥1 at the genus level) for *Mycobacterium tuberculosis* complex; (7) SDSMRN ≥3 for *Nocardia* spp. as a positive result.

### Statistical analysis

SPSS version 22.0 software (IBM Corp., Armonk, NY, USA) was utilized for data analysis. The independent t-test was performed to compare the differences in counting data between the two groups. The chi-squared test was used to evaluate the categorical variables. A *P*-value below 0.05 was regarded as a significant difference.

### Data availability

Sequencing data were deposited to the National Genomics Data Center under accession number CRA016496. The authors declare that the main data supporting the findings are available within this article. The other data generated and analyzed for this study are available from the corresponding author upon reasonable request.

## Results

### Clinical characteristics

In this study of 90 patients, 46 were identified as HIV-positive. Within this HIV-infected cohort, a significantly higher proportion were male compared to females, marking a clear contrast with the non-HIV group. Furthermore, the ages of patients in the HIV group were significantly lower than those in the non-HIV group. Across all these patients with pulmonary infections, the predominant symptoms were fever, cough, and expectoration, with no significant differences between the two groups. Patients infected with HIV have significantly lower counts of CD4 and the CD4/CD8 ratio compared to patients without HIV. In addition, the occurrence of underlying diseases was significantly greater in the non-HIV group compared to the HIV-infected group, with hypertension being the most common among these conditions (**Table 1**).

**Table 1:** Basic characteristics.

	Total (n=90)	HIV (n=46)	Non-HIV (n=44)	*P*-value
Sex				
Male	67(74.4%)	44(95.7)	23(52.3%)	1.6E-6
Female	23(25.6%)	2(4.3%)	21(47.7%)	
**Age (**Median[IQR])	52(40-63.5)	43.5(33.25-53)	59(51.75-69)	9.3E-7
Clinical characteristics				
Fever	82(91.1%)	41(89.1%)	41(93.2%)	0.7
Cough	69(76.7%)	35(76.1%)	34(77.3%)	1
Expectoration	53(58.9%)	23(50%)	30(68.2%)	0.1
Diarrhea	16(17.8%)	11(23.9%)	5(11.4%)	0.2
Immunity index				
CD4 [Median(IQR)]	128.5 (13.725-484.75)	13.75 (6.1-35.25)	511.5 (315-759.5)	3.3E-12
CD4/CD8 [Median(IQR)]	0.225 (0.0525-1.93)	0.055 (0.02-0.1)	2.02 (0.84-3.205)	2.2E-13
Comorbidities				
Hypertension	16(17.8%)	2(4.3%)	14(31.8%)	7.2E-4
Coronary heart disease	2(2.2%)	0	2(4.5%)	0.2
Diabetes	7(7.7%)	0	7(15.9%)	5.1E-3
Tumor	7(7.7%)	0	7(15.9%)	5.1E-3
Interstitial lung disease	1(1.1%)	0	1(2.3%)	0.5

### Pathogen profiles of HIV group and non-HIV group

Two physicians independently determined the causative pathogens for each patient based on the results of mNGS and CTM, as well as the patient’s treatment strategies, and prognosis. In cases of disagreement, a third physician provided the final determination. According to the final clinical diagnosis results, a total of 37 pathogens were observed in this study, including 21 bacteria, 5 fungi, 5 viruses, 5 mycobacteria, and 1 mycoplasma. We found a notably higher incidence of fungal and viral infections among HIV-infected patients compared to those without HIV infection (**Table 2**).

**Table 2:** Pathogen types in the BALF from the HIV and non-HIV infection groups.

Pathogen type	HIV infection group	Non-HIV infection group	*P*
Fungi	91.3% (42/46)	20.5% (9/44)	3.7E-12
Viruses	67.4% (31/46)	6.8% (3/44)	1.1E-9
Bacteria	34.8% (16/46)	47.7% (21/44)	0.3

BALF, bronchoalveolar lavage fluid.

**Figure 1:**
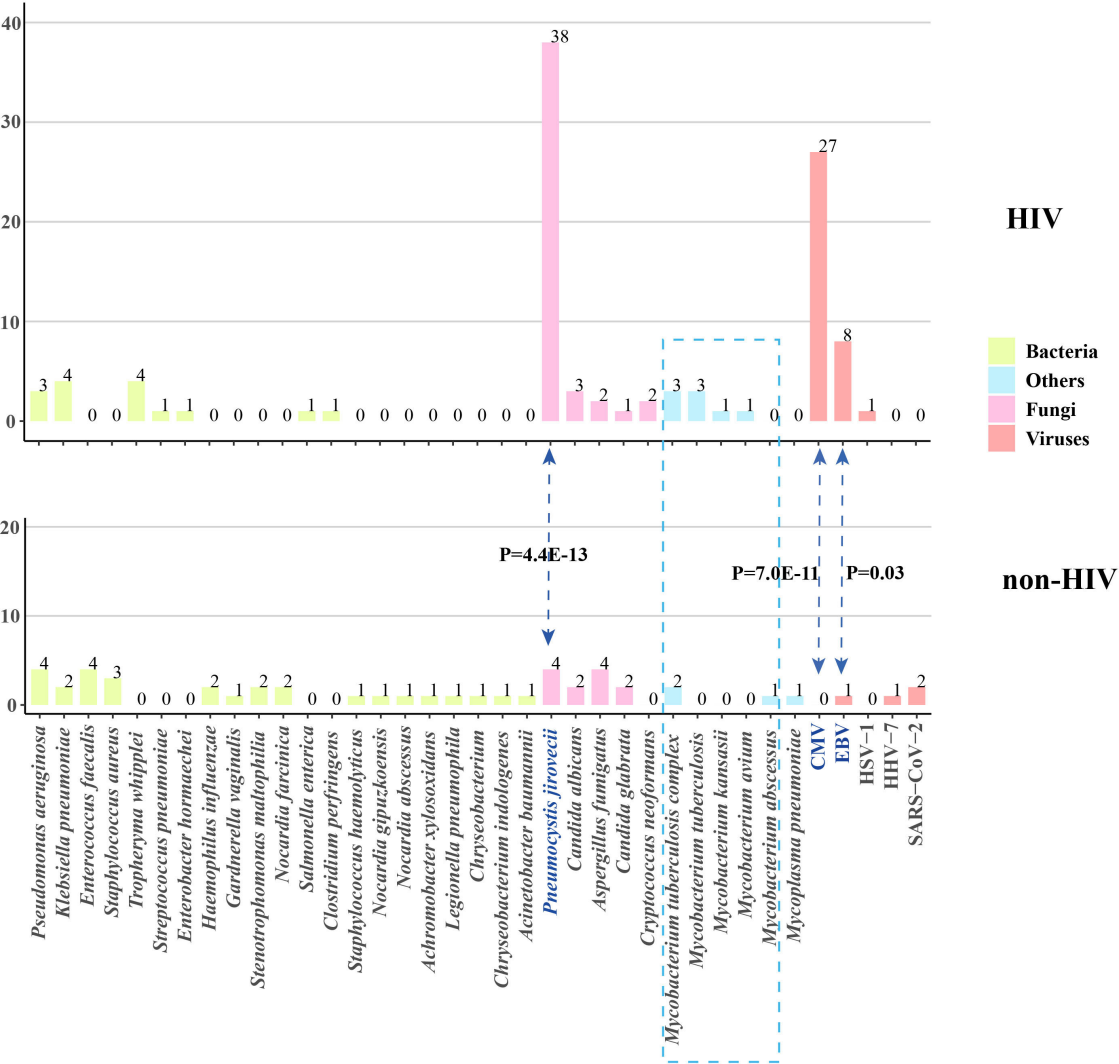
Pathogen profiles of patients with and without HIV infection. CMV, cytomegalovirus; EBV, Epstein-Barr virus; HSV, herpes simplex virus; HHV, human herpes virus; HIV, human immunodeficiency virus.

The pathogen spectrum in patients from both the HIV and non-HIV groups was shown in **Figure 1**. The results indicated that the primary reason for the significantly higher rate of fungal and viral infections in HIV patients was attributed to *Pneumocystis jirovecii*, cytomegalovirus (CMV), and Epstein-Barr virus (EBV). The prevalence of *P. jirovecii* in HIV patients was as high as 82.6% (38/46), which was much higher than in the non-HIV group. For the rarely pathogenic agents CMV and EBV, their potential for opportunistic infection should not be underestimated due to the unique characteristics of HIV patients. The occurrence rates of other pathogens do not differ between the two groups, but it is noteworthy that the frequency of *Mycobacteria* in HIV patients is also higher than that in non-HIV patients, although the difference is not statistically significant.

### Comparison of mNGS with CTM

**Figure 2:**
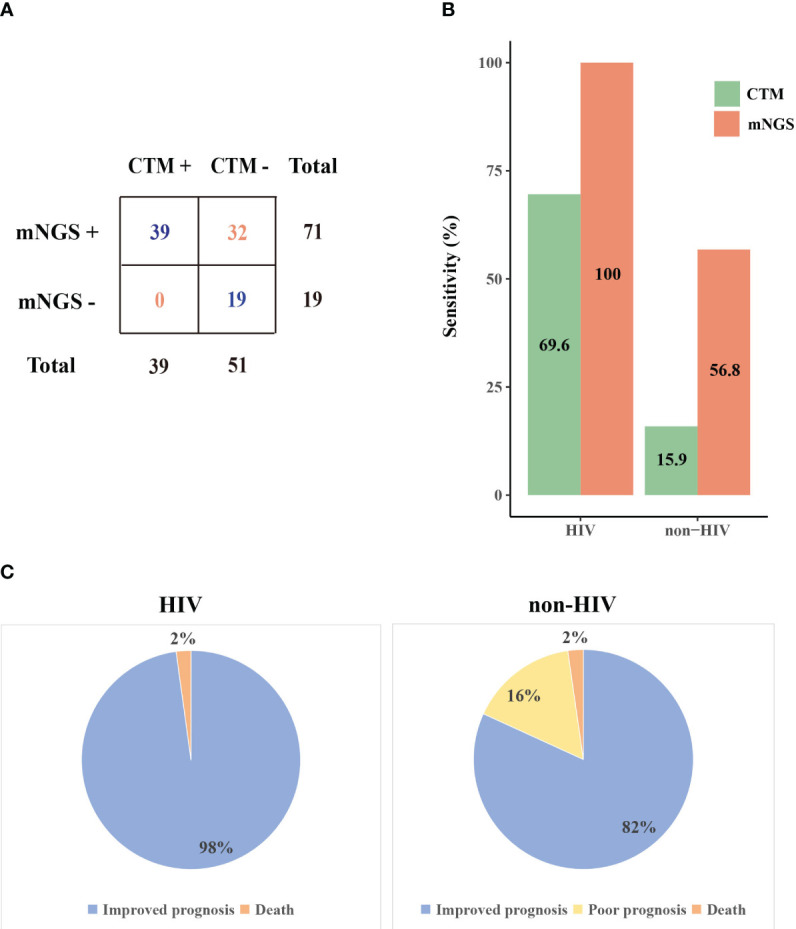
The performance of mNGS and CTM in patients with different prognoses. **(A)** Comparison of mNGS and CTM detection **(B)** The sensitivity of mNGS and CTM in HIV and non-HIV group **(C)** Prognosis of patients in HIV and non-HIV group. CTM, conventional methods.

We further compared the mNGS detection results with CTM (**Figure 2A**). The sensitivity of mNGS reached 78.9% (71/90), which is markedly superior to that of CTM (39/90, *P*=1.5E-8). The superiority of mNGS over CTM was demonstrated in both the HIV and non-HIV groups (**Figure 2B**). The negative results of mNGS included 10 patients with no microorganisms detected, and 9 cases where the detected pathogen was excluded as the causative agents. All of these 19 patients were not infected with HIV, and their CTM also showed negative results.

**Figure 3:**
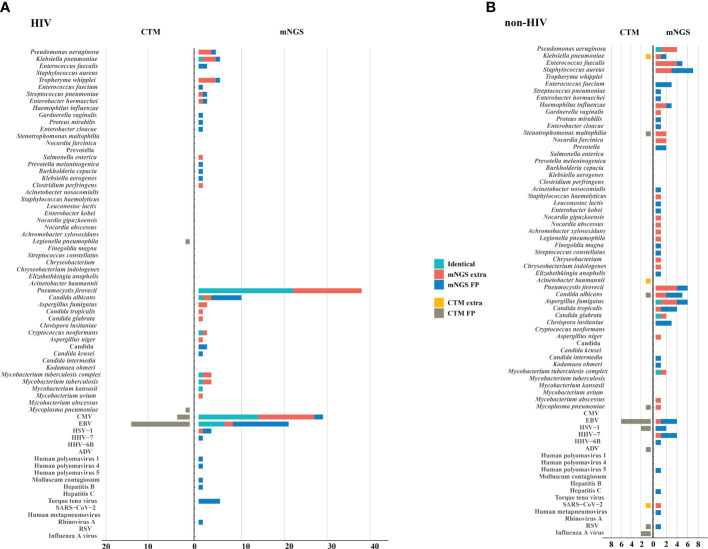
Potential pathogens identified by mNGS and CTM. **(A)** HIV group; **(B)** non HIV group. The pathogens consisted of identical detection (the green) of mNGS to CTM, extra detection (the red) of mNGS, false positive detection (the blue) of mNGS, extra detection (the yellow) of CTM, false positive detection (the grey) of CTM. The false positive detection of mNGS represents those microorganisms were detected only by mNGS and diagnosed as non-causitive pathogens. The false positive detection of CTM represents those microorganisms were detected only by CTM and diagnosed as non-causitive pathogens. CMV, cytomegalovirus; EBV, Epstein-Barr virus; HSV, herpes simplex virus; HHV, human herpes virus; HIV, human immunodeficiency virus; ADV, adenovirus; RSV, respiratory syncytial virus.

The microorganisms identified by mNGS and CTM was depicted in **Figure 3**. In HIV patients, CTM missed many cases of *P. jirovecii* (n=16) and CMV (n=13), which were all detected by mNGS. Similarly, in non-HIV patients, mNGS detected bacterial and fungal infections that CTM failed to identify. mNGS only missed 3 pathogenic agents detected by CTM, including one case of *Klebsiella pneumoniae*, one case of *Acinetobacter baumannii*, and one case of SARS-CoV-2. In this study, two patients died due to multi-organ failure, including one HIV-positive individual and one non-HIV-positive individual. The vast majority of the remaining patients attained improved prognoses through treatment strategies informed by pathogen detection results (**Figure 2C**). Thus, it can be seen that mNGS detection can greatly reduce the rate of missed diagnoses due to the insufficient sensitivity of CTM.

### The performance of tNGS

**Figure 4:**
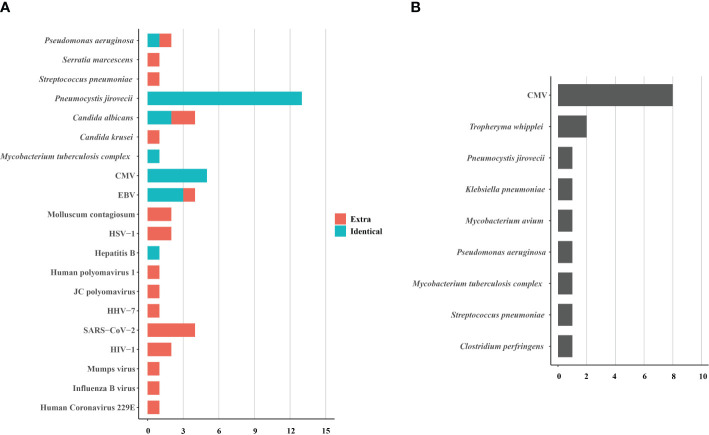
The pathogens identified **(A)** and not identified **(B)** by tNGS in 15 HIV-infected patients. The pathogens consisted of identical detection (the green) of tNGS to mNGS and extra detection (the red) of tNGS. CMV, cytomegalovirus; EBV, Epstein-Barr virus; HSV, herpes simplex virus; HHV, human herpes virus; HIV, human immunodeficiency virus.

BALF samples from 15 HIV patients underwent concurrent tNGS detection to assess its performance. Among them, tNGS identified the primary causative pathogens in 13 patients, achieving a consistency rate of 86.7% (13/15) compared to mNGS. tNGS identified some additional pathogens, mainly viruses, compared to mNGS (**Figure 4A**). However, the pathogenicity of most of these viruses was not considered. Additionally, tNGS successfully identified all causative pathogens in 3 cases. In other cases, CMV was the main pathogen that tNGS did not detect. (**Figure 4B**). Hence, the diagnostic potential of tNGS for pulmonary infections in HIV patients is promising.

## Discussion

It is crucial to identify pathogens and perform accurate treatment regimens on patients with pulmonary infection. The conventional detection methods include sputum smear, culture, and other molecular tests, but they have low diagnostic rates, which may delay the timing of treatment (Caetano Mota et al., 2012). In this study, we performed mNGS analysis on the BALF from HIV-infected and non-HIV-infected patients with pulmonary infection and found significant differences in the pathogen profiles between the two study groups. tNGS detection was simultaneously performed on BALF from 15 HIV-infected patients to observe the clinical utility of tNGS in HIV-infected patients.

In this study, patients in the non-HIV group were found to have a higher prevalence of bacterial infections, while HIV patients were more commonly infected with fungi and viruses. Particularly notable was the significantly higher proportion of *P. jirovecii*, CMV, EBV, and tuberculosis in HIV-infected patients compared to those without HIV infection. This indicated that they had multi-pathogenic pneumonia and were at a higher risk of developing opportunistic infections. Opportunistic infections frequently occur in individuals with acquired immune deficiency (Shi et al., 2022), and the current study showed that the HIV-positive patients were infected with more than one type of pathogens, such as *P. jirovecii*, *M. tuberculosis*, and nontuberculous mycobacteria, as well as viruses, suggesting that they were susceptible to developing a mixed infection. Previous research has revealed that HIV-infected individuals with a lower CD4^+^ T-cell count and CD4^+^/CD8^+^ ratio are prone to pneumonia (Xie et al., 2023). Therefore, monitoring CD4^+^ lymphocytes with mNGS analysis is a favorable detection method for HIV-infected patients with suspected lung infection. Moreover, mNGS can identify the underlying pathogenic germs as well as vulnerable individuals like the HIV-infected population.

This study revealed that the constituent ratio of *P. jirovecii* ranked first among the HIV-infected patients and was higher than that among the non-HIV-infected patients. This finding is consistent with the previous studies demonstrating that this pathogen is more likely to occur in immunosuppressed hosts (Souza Lopes et al., 2016). *P. jirovecii* can aggravate lung exudation and increase the mortality of infected patients, especially those with an impaired immune function (Cheng et al., 2023). Therefore, early detection of such life-threatening bacteria can help clinicians determine the right treatment strategy to prevent infected patients from developing severe pneumonia.

Different hospitals have distinct detection capabilities and methods, which can greatly affect the detection rate of pathogens. In China, smear staining remains the primary detection method for *P. jirovecii* (Li et al., 2020). However, there is no unified operation, quality-control process, or diagnostic standard, which brings great diagnostic difficulties to clinicians. Over the years, culture has been used as the gold standard for the diagnosis of *M. tuberculosis* and nontuberculous mycobacteria, but the bacterial culture requires harsh conditions for operators and laboratories (Jain et al., 2017). In addition, its procedure takes a long time, which cannot be fed back to clinicians in time. Moreover, the culture method shows low sensitivity. With the advancement of mNGS, the detection rates of *M. tuberculosis* and nontuberculous mycobacteria have been greatly increased, especially for drug-resistant strains, indicating that a large proportion of patients can receive effective, timely, and accurate treatment. Serum markers and immunologic assays also showed low specificity in the detection of *Cryptococcus*, various viruses, and other pathogens (Jarvis et al., 2014). Additionally, non-HIV-infected people with community-acquired or nosocomial pneumonia had a very low chance of being detected with true pathogenic pathogens when only CTM were used, and the accuracy rate of sputum smear and culture methods was less than 50% (Caetano Mota et al., 2012).

Many studies have shown that mNGS is an irreplaceable method for identifying pathogens in complex infections. Compared with CTM, mNGS shows higher sensitivity because it can simultaneously detect multiple pathogens and mutated genes as well as requires a small amount of sample, which can help achieve a precise anti-infection treatment (Morganti et al., 2019; Pourahmadiyan et al., 2022). Although the adoption rate of mNGS remains low in resource-poor areas of the world, it is expected to be extensively used for the detection of complex infectious diseases with the continuous progress of mNGS technology and the significant decline in its cost.

Compared to the high economic burden of mNGS, tNGS is more economically feasible for clinical application. tNGS is a method that combines gene-targeted PCR amplification and high-throughput sequencing, and its application value for pathogen detection in clinical settings has been evaluated in several literature studies (Mertes et al., 2011; Gaston et al., 2022). tNGS can rapidly identify pathogens within 24-48 hours and complete the detection of drug-resistant genes and virulence genes (Onda et al., 2018). However, its applicability to patient populations is relatively limited. If the patient presents with fever of unknown origin, unclear etiology, and uncertain infectious pathogens, clinicians may require a broader range of testing to assist in diagnosis, especially for some rare and specific infectious pathogens. However, for some populations, such as HIV-infected individuals, opportunistic infectious pathogens are predominant. The tNGS detection range can almost meet the needs of the majority of patients, and it is more economical, making it more widely applicable in clinical practice.

### Limitation

This study had some limitations. First, it was a small-sample, single-center research study, which might limit the accuracy of the study. Further research should be performed with more samples from distinct areas. Second, there is no unified detection process for mNGS, and we did it according to the published literature, which might cause false-positive or false-negative results. So, the data interpretation was carried out by three experienced laboratory personnel to lower the result bias as much as possible. Finally, due to the limited budget, not all HIV patients underwent tNGS testing, and its performance still requires further research.

## Conclusions

It is important to perform mNGS or tNGS in immunosuppressed patients combined with pulmonary infection since these methods can quickly and precisely reveal pathogens to help determine an effective anti-infective regimen. Therefore, both mNGS and tNGS should be promoted in the clinic for better patient outcomes.

## Data Availability

The data presented in the study are deposited in the China National Center for Bioinformation - National Genomics Data Center - Genome Sequence Archive, submission numbers CRA016496 and CRA017709.
